# Forschung für die Praxis: Bullöse Autoimmundermatosen unter Immuncheckpoint‐Inhibitoren

**DOI:** 10.1111/ddg.15638_g

**Published:** 2025-04-08

**Authors:** Jasper N. Prüßmann, Wiebke Prüßmann, Christian D. Sadik

**Affiliations:** ^1^ Klinik für Dermatologie Allergologie und Venerologie Universität zu Lübeck Universitätsklinikum Schleswig‐Holstein, Lübeck

**Keywords:** Autoimmundermatose, Immuncheckpoint, Immunbedingte unerwünschte Wirkung, Pemphigoid, autoimmune dermatosis, immune checkpoints, immune related adverse event, pemphigoid

## Abstract

Immuncheckpoint‐Rezeptoren und ‐Liganden wie das *cytotoxic T‐lymphocyte‐associated Protein 4* (CTLA‐4), *programmed death‐1* (PD‐1) und *ligand‐1* (PD‐L1) werden sowohl auf Immun‐ als auch auf nicht‐Immunzellen in großem Umfang exprimiert und dienen der Feinabstimmung des Aktivierungsniveaus von Immunzellen, wodurch Immunreaktionen ermöglicht, verhindert oder beendet werden. Die Blockade von CTLA‐4, PD‐1 oder PD‐L1 durch Checkpoint‐Inhibitoren (CI) entfesselt Immunreaktionen und ist zu einer tragenden Säule bei der Behandlung verschiedener Krebsarten geworden. Die Induktion autoinflammatorischer, jedoch unspezifischer Gewebeschäden in verschiedenen Organen wird als immunbedingte unerwünschte Wirkung (*immune related adverse event*, irAE) bezeichnet, eine klassentypische Nebenwirkung der CI, die den Abbruch der Immuntherapie erfordern kann. Unter den häufig auftretenden Hautausschlägen können CI, die auf die PD‐L1/PD‐1‐Achse abzielen, bei etwa 0,3% bis 0,6% der behandelten Patienten das durch IgG‐Autoantikörper und Granulozyten hervorgerufene bullöse Pemphigoid (BP) auslösen. Die Pathogenese des BP erfordert eine komplexe zelluläre Entzündungsreaktion nach Bindung von Anti‐BP180‐Autoantikörpern an die dermo‐epidermale Junktion. Die Prävalenz von Autoantikörpern gegen BP180 bei gesunden Blutspendern von etwa 0,52% entspricht der Prävalenz von irBP bei behandelten Krebspatienten, was die potenzielle Bedeutung der PD‐1‐vermittelten Regulierung der Entzündungsreaktion im Gewebe beim spontanen BP unterstreicht. Wenn unter der Checkpoint‐Inhibitor‐Therapie Hautreaktionen auftreten, sollten Biopsien entnommen und durch histopathologische und direkte Immunfluoreszenzmikroskopie untersucht werden.

## KLINISCHE RELEVANZ

„Immuncheckpoints“, das heißt co‐stimulatorische und co‐inhibitorische Rezeptoren sowie ihre Liganden, werden weit verbreitet auf Immun‐ und nicht‐Immunzellen exprimiert und spielen eine zentrale Rolle als Regulatoren von Immunreaktionen. Sie sind entscheidend für die Feinabstimmung des Aktivierungsniveaus von Immunzellen, indem sie Immunreaktionen ermöglichen, verhindern oder beenden. Ihre Funktion ist von zentraler Bedeutung für die Entscheidung, ob eine Immunreaktion ausgelöst wird oder ob Toleranz und Gewebshomöostase aufrechterhalten werden sollen.

Die Immuncheckpoints *programmed death 1* (PD‐1) und sein *ligand 1* (PD‐L1) sowie *cytotoxic T‐lymphocyte‐associated Protein 4* (CTLA‐4) sind die bekanntesten Checkpoint‐Inhibitor‐Moleküle (CIM) und kontrollieren direkt den Aktivierungsgrad von T‐Zellen.

*Cytotoxic T‐lymphocyte‐associated Protein 4* (CTLA‐4) ist ein co‐inhibitorischer und kompetitiver Ligand des co‐stimulatorischen T‐Zell‐Rezeptors CD28, der das zweite Signal zur T‐Zell‐Rezeptor (TCR)‐Aktivierung vermittelt. Durch die Bindung und Blockierung von CD80 und CD86, den Liganden von CD28, die von antigenpräsentierenden Zellen (APC) exprimiert werden, schränkt CTLA‐4 die Stimulation naiver T‐Zellen ein.^1^ Nach der Aktivierung naiver T‐Zellen wird CTLA‐4 verstärkt exprimiert und konstitutiv auf regulatorischen T‐Zellen exprimiert. Ein CTLA‐4‐Defekt führt zu einer lymphoproliferativen Erkrankung, die mit durch Autoantikörper vermittelten Zytopenien, organspezifischer Autoimmunität, Lymphadenopathie und Splenomegalie, lymphozytärer Infiltration nicht‐lymphoider Organe und B‐Zell‐Erschöpfung und dadurch bedingter Hypogammaglobulinämie mit erhöhter Infektionsanfälligkeit einhergeht.^2^ PD‐1 wird durch kontinuierliche TCR‐Signale induziert. Sein Ligand PD‐L1 wird im Ruhezustand und nach Aktivierung von verschiedenen Immun‐ und Nicht‐Immun‐Zellen exprimiert.^1^ Daher dient PD‐1 den T‐Zellen bei fast allen Zellkontakten als Immuncheckpoint. Bislang wurde ein Defekt von PD‐1 nur bei einem einzigen Menschen beschrieben. Dieser Patient litt unter Autoimmunreaktionen in verschiedenen Organen und einer Störung des Immunschutzes gegen Tuberkulose. Bei ihm wurde eine stark gestörte Lymphozytenhomöostase mit einer Verarmung und Dysfunktion spezifischer T‐ und natürlicher Killerzellen und einer Vermehrung unspezifisch aktivierter, Zytokin freisetzender T‐Zellen beobachtet.^3^ Zusammengefasst modulieren Immuncheckpoints die T‐Zell‐Rezeptor‐Signalübertragung durch das Hinzufügen stimulierender oder inhibitorischer Signale. Dadurch werden co‐inhibitorische Rezeptoren und ihre Liganden zu zentralen Regulatoren der T‐Zell‐vermittelten Immunantwort.

Die Blockade von CTLA‐4, PD‐1 oder PD‐L1 durch Checkpoint‐Inhibitoren (CI), die Immunreaktionen entfesseln, ist zu einer tragenden Säule bei der Behandlung verschiedener Krebsarten geworden. Die Auslösung autoinflammatorischer, jedoch unspezifischer Gewebeschäden in verschiedenen Organen, die vor allem die Haut, den Darm, die Lunge oder die endokrinen Drüsen betreffen, werden als immunbedingte unerwünschte Wirkungen (*immune related adverse events*, irAE) bezeichnet, eine klassentypische Nebenwirkung von CI.^4^, ^5^ Obwohl das Auftreten von irAE häufig mit besserem Ansprechen auf die Immuncheckpoint‐Inhibitor‐Behandlung einhergeht, kann dies leider auch das Absetzen von CI erfordern.^6^ Checkpoint‐Inhibitoren mit Wirkung auf die PD‐L1/PD‐1‐Achse lösen bei etwa 0,3% bis 0,6% der behandelten Patienten eine spezifische, durch IgG‐Autoantikörper und Granulozyten hervorgerufene entzündliche Erkrankung der Haut aus, das bullöse Pemphigoid (BP).^7^, ^8^, ^9^, ^10^, ^11^

Beim BP binden Autoantikörper Strukturproteine des Adhäsionskomplexes an der dermo‐epidermalen Junktion (DEJ), nämlich BP180 (Kollagen Typ XVII) und/oder BP230 (Dystonin, Teil der Hemidesmosomen). Dies leitet die Effektorphase der Krankheit mit einer entzündlichen Kaskade aus Komplementaktivierung, Anlockung von Granulozyten und Freisetzung von Proteasen und anderen lytischen Verbindungen ein, die schließlich zur Zerstörung der DEJ und Blasenbildung führt.^12^ Die Patienten sind zum Zeitpunkt der Krankheitsmanifestation meist über 75 Jahre alt, ohne starke Geschlechtspräferenz, und die Gesamtprävalenz des BP steigt bei über 80‐Jährigen auf 200 Fälle pro Million pro Jahr.^13^ Mit der Krankheitsmanifestation scheinen häufig neurologische und psychiatrische Störungen wie Parkinson, Demenz und uni‐ oder bipolare Störungen^14^ sowie bestimmte Medikamente wie Diuretika, Antipsychotika und Dipeptidyl‐Peptidase‐IV‐Hemmer (Gliptine) assoziiert zu sein.^12^, ^13^ Typischerweise zeigen die Hautläsionen einen Verlauf von Erythem über urtikarielle Plaque, Blase und Erosion bis hin zu hyper‐ oder hypopigmentierten reepithelisierten Maculae ohne Narbenbildung. Diese Veränderungen treten gleichzeitig in unterschiedlichen Stadien und an verschiedenen Körperregionen auf. Der bullöse Pemphigoid (BP) ist jedoch eine klinisch heterogene Erkrankung, da alle Stadien der Hauteruptionen auch isoliert auftreten können. Ein charakteristisches Symptom bei den meisten BP‐Patienten ist starker Juckreiz. Andererseits kann Juckreiz beim sogenannten prämonitorischen BP auch das einzige Symptom sein.^13^ Beim prämonitorischen, klassischen bullösen und nichtbullösen Pemphigoid bestehen mehrere klinische Differenzialdiagnosen, darunter Skabies, bullöse Arzneimittelreaktionen oder Ekzeme, die jeweils spezifische Therapien erfordern. Der Goldstandard für die Diagnose des BP ist eine periläsionale Biopsie mit direkter Immunfluoreszenzfärbung von gewebegebundenen Autoantikörpern, die eine lineare Ablagerung an der Basalmembran zeigen. Eine läsionale Biopsie zur histopathologischen Untersuchung ist notwendig, um Differenzialdiagnosen auszuschließen. Darüber hinaus ist der Nachweis von zirkulierenden Autoantikörpern im Blut der Patienten erforderlich, um das Zielantigen zu identifizieren, wodurch die verschiedenen Pemphigoid‐Erkrankungen unterschieden und die Aktivität der Krankheit überwacht werden können. Die Therapie des BP basiert auf Immunsuppressiva und umfasst die systemische Gabe von Kortikosteroiden und/oder die (ganzkörperliche) topische Behandlung mit stark wirksamen Kortikosteroiden.^15^ Die Mortalität sank von 70% während des ersten Auftretens eines BP in der Zeit vor der Behandlung mit Immunsuppressiva auf eine 2–3fach erhöhte Gesamtmortalität im Vergleich zu alters‐ und geschlechtsgleichen Kontrollgruppen bei adäquater Behandlung.^13^ Mortalität ist häufig mit Infektionen verbunden.

Die Spezifität und Häufigkeit des durch Checkpoint‐Therapien ausgelösten BP als Nebenwirkung deutet darauf hin, dass PD‐L1/PD‐1 eine Rolle in der Pathophysiologie des BP spielen könnten. Obwohl PD‐L1 und PD‐1 weithin exprimiert werden, einschließlich durch Granulozyten, und auch in der entzündeten Haut von BP‐Patienten nachweisbar sind, sind ihre Funktion bei Autoantikörper‐ und Granulozyten‐gesteuerten Immunreaktionen sowie die Rolle von T‐Zellen beim BP insgesamt bisher nur unzureichend verstanden.^16^

## HYPOTHESE

**ABBILDUNG 1:**
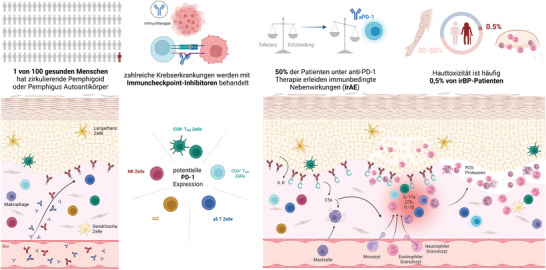
Vermutete Pathophysiologie des immuntherapiebedingten bullösen Pemphigoids (irBP, *immunotherapy related bullous pemphigoid*). Etwa 1% der gesunden Menschen sind asymptomatische Träger von zirkulierenden, möglicherweise in der Haut gebundenen Pemphigoid‐ oder Pemphigus‐assoziierten nicht‐pathogenen Autoantikörpern. In gesunder Haut sind nur wenige residente Immunzellen vorhanden. Wir stellen die Hypothese auf, dass CD8^+^ TRM‐Zellen, CD4^+^ TRM‐Zellen, γδ‐T‐Zellen, angeborene lymphatische Zellen (ILC) und natürliche Killerzellen (NK) unter normalen Bedingungen PD‐1 auf ihrer Zelloberfläche exprimieren könnten. Durch pharmakologische PD‐1‐Hemmung könnten diese Zellen aktiviert werden, Zytokine freisetzen und Fc‐Rezeptor‐unabhängige Vorgänge auslösen, die zur Freisetzung von Interleukin (IL)‐8 aus basalen Keratinozyten führen. Komplement wird aktiviert und an der dermo‐epidermalen Grenzfläche (DEJ) gebunden, was zur Degranulation von Mastzellen führt. Chemokin‐Gradienten (IL‐17a, LTB4, IL‐1β) fördern die Infiltration der oberen Dermis durch Effektorzellen wie Monozyten, Neutrophile und Eosinophile. Granulozyten an der DEJ setzen Proteasen und reaktive Sauerstoffspezies (ROS) frei, die zur subepidermalen Blasenbildung bei 0,3% und 0,6% der mit Anti‐PD‐1‐Checkpoint‐Inhibitoren behandelten Patienten führen und somit ein irBP entwickeln (erstellt mit BioRender.com).

Die gelegentliche Manifestation von BP unter PD‐1/PD‐L1‐Hemmung deutet darauf hin, dass die PD‐1/PD‐L1‐Achse der Entstehung und Schwere des spontan auftretenden BP entgegenwirken kann. Möglicherweise geschieht dies auf Ebene eines Toleranzverlusts gegenüber BP180, was zur Bildung pathogener Antikörper führt, sowie auf der Ebene der Induktion und Gegenregulation von Gewebeentzündungen. Bei gesunden Blutspendern (Altersbereich 18–69 Jahre) beträgt die Gesamtprävalenz der häufigsten hautspezifischen Autoantikörper (Anti‐Desmoglein 1, ‐Desmoglein 3, ‐BP180, ‐BP230) etwa 1% (Abbildung 1).^17^ Während Desmoglein‐spezifische Antikörper durch bloße Bindung ihrer Antigene zur Akantholyse führen, erfordert die Pathogenese von Pemphigoiderkrankungen nach Antikörperbindung eine komplexe zelluläre Entzündungsreaktion. Es ist wichtig festzuhalten, dass erstens Pemphiguserkrankungen bisher nur in Einzelfällen als irAE beschrieben wurden,^18^ was darauf hindeutet, dass die Entwicklung pathogener Autoantikörper vermutlich eher nicht durch eine CTLA‐4‐ oder PD‐1/PD‐L1‐Immuntherapie ausgelöst wird. Zweitens liegt die Prävalenz von Autoantikörpern gegen BP180 bei gesunden Blutspendern bei etwa 0,52%, wenngleich sie bei älteren Menschen vermutlich höher ist, aber immer noch nahe an der Prävalenz von irBP bei behandelten Krebspatienten,^17^, ^19^ was die potenzielle Bedeutung der PD‐1‐vermittelten Regulierung der Gewebeentzündung bei spontanem BP unterstreicht.

Die IgG‐Produktion durch B‐Zellen in den B‐Zell‐Follikeln der Lymphknoten hängt von der Hilfe spezifischer T‐Zellen ab, die als T‐Follikel‐Helferzellen (*T follicular helper cells*; Tfh) bezeichnet werden. Dies deutet darauf hin, dass die Produktion von Autoantikörpern in der präklinischen Phase einer Krankheit bereits die Aktivierung autoreaktiver T‐Zellen erfordert. Im Vergleich zur PD‐1/PD‐L1‐Hemmung wurde BP im Zusammenhang mit einer Anti‐CTLA‐4‐Monotherapie nur in wenigen Fallberichten beschrieben, obwohl irAE bei der CTLA‐4‐Hemmung häufiger auftreten und oft schwerer verlaufen als bei der PD‐1/PD‐L1‐Hemmung.^6^, ^20^ Während die Hemmung von CTLA‐4 wahrscheinlich die Aktivierung von naiven, selbstreaktiven T‐Zellen durch APC verstärkt, könnte die Unterbrechung der PD‐1/PD‐L1‐Achse in erster Linie die periphere Toleranz durch die Aktivierung von Effektor‐ und Gedächtnis‐T‐ Zelluntergruppen stören.^1^ Die Rolle von T‐Zellen und PD‐1 in der Pathogenese von BP ist noch nicht vollständig geklärt, aber Tfh und hautresidente Gedächtnis‐T‐Zellen exprimieren PD‐1 und sind möglicherweise beteiligt.^21^ Da fast die Hälfte der Patienten unter der Checkpoint‐Therapie ekzematöse oder lichenoide Hautausschläge entwickelt (Abbildung 1), stellen wir die Hypothese auf, dass bereits vorhandene, an die Haut gebundene Anti‐BP180‐Autoantikörper die Entzündungsreaktion, die durch aktivierte PD‐1‐exprimierende (autoreaktive) T‐Zellen in der Haut ausgelöst wird, in eine von Granulozyten dominierte Entzündung mit Zerstörung der dermo‐epidermalen Grenzfläche lenken. Um diese Hypothese zu prüfen, untersuchen wir die Rolle der PD‐1‐Hemmung speziell in der Effektorphase der durch Autoantikörper ausgelösten und von Granulozyten gesteuerten Immunantwort bei Pemphigoiderkrankungen.

## FORSCHUNG FÜR DIE KLINISCHE PRAXIS

Wir haben vorläufige Daten aus einem Mausmodell der Effektorphase von Pemphigoid‐Erkrankungen gewonnen, die darauf hindeuten, dass PD‐1 und PD‐L1 der Hautentzündung in diesem Modell entgegenwirken. So wurde die Hautentzündung verschlimmert, wenn PD‐1 genetisch ausgeschaltet oder pharmakologisch gehemmt wurde. Diese Ergebnisse unterstützen die Annahme, dass eine Behandlung mit Checkpoint‐Inhibitoren die Entstehung von bullösem Pemphigoid (BP) auf der Ebene der Effektorphase fördern kann. Darüber hinaus könnte die Aktivierung von PD‐1/PD‐L1 ein vielversprechender therapeutischer Ansatz zur Behandlung des spontanen BP sein. Wir untersuchen dieses Behandlungskonzept derzeit im Rahmen eines von der Deutschen Dermatologischen Gesellschaft (DDG) und der Arbeitsgemeinschaft Dermatologische Forschung (ADF) finanzierten Forschungsprojekts.

Die Bedeutung der PD‐1‐Signalübertragung für die Aufrechterhaltung der peripheren Toleranz durch Begrenzung der Aktivität autoreaktiver T‐Zellen in der Haut wurde auch bei lichenoider irAE festgestellt. Damo et al. entwickelten ein Antigen‐Modellsystem, bei dem die Epidermis der Maus ein T‐Zell‐spezifisches Modellantigen exprimiert. Sie zeigten, dass, obwohl spezifische CD8‐T‐Zellen für dieses Antigen einen Effektor‐Phänotyp erwerben, keine Pathologie beobachtet wird. Diese Zellen akkumulieren in der Dermis, erreichen aber nicht ihre epidermalen Zielzellen, weil die PD‐1‐ Signale diese Zellen daran hindern, in die Epidermis zu wandern, Zytokine freizusetzen und dadurch pathologische Prozesse auszulösen. Patienten, die unter einer durch PD‐1‐Therapie induzierten lichenoiden Hauteruption leiden, tragen tatsächlich klonal expandierte Effektor‐CD8‐T‐Zellen in der läsionalen und nichtläsionalen Haut.^22^

## SCHLUSSFOLGERUNGEN FÜR DIE KLINISCHE PRAXIS

Das Verstehen der Funktion der Immuncheckpoints und ihre gezielte Nutzung stellen einen wichtigen Fortschritt bei der Behandlung bösartiger Erkrankungen dar. Die neue Klasse immunbedingter unerwünschter Wirkungen betrifft fast die Hälfte der behandelten Patienten. Defekte der Immuncheckpoints bei menschlichen Patienten sind zwar nur in Fallstudien dokumentiert, haben aber dennoch dazu beigetragen, ihre Funktion besser zu verstehen. Obwohl die inhibitorische Checkpoint‐Therapie bereits seit etwa einem Jahrzehnt klinisch eingesetzt wird, ist der CTLA‐4‐Agonist Abatacept immer noch der einzige CIM‐Aktivator auf dem Markt, der zur Behandlung von Autoimmunerkrankungen eingesetzt wird. Dieser birgt jedoch das Risiko schwerer, potenziell tödlicher Infektionen. Die Entwicklung von PD‐1‐Agonisten zur Behandlung von Autoimmunkrankheiten war jedoch bisher nicht erfolgreich, was darauf zurückzuführen sein könnte, dass PD‐L1 im Überfluss vorhanden ist und für periphere Toleranz sorgt, wahrscheinlich sogar bei Autoimmunkrankheiten.^23^ Die vielfältigen Möglichkeiten, wie Patienten mit irAE der Haut auf eine Checkpoint‐Therapie reagieren, zeigen, dass der individuelle Kontext die Richtung der Entzündung stark beeinflusst, die von häufigen pruritischen, lichenoiden, ekzematösen, psoriasiformen bis hin zu seltenen humoralen und granulozytären dominierten bullösen Immunreaktionen reicht.^24^ Die Identifikation zentraler Zytokine, die diesen unterschiedlichen Reaktionen zugrunde liegen, ist entscheidend, um gezielte Behandlungen von immunbedingten Nebenwirkungen (irAE) zu entwickeln, ohne den therapeutischen Nutzen der Krebsimmuntherapie zu beeinträchtigen, und um alternative Therapien für Autoimmunerkrankungen jenseits von Immuncheckpoint‐Agonisten oder allgemeinen Immunsuppressiva zu ermöglichen. Treten während der Immuncheckpoint‐Inhibitor‐Therapie Hautausschläge auf, sollte die Entnahme von Biopsien aus läsionaler und periläsionaler Haut in Erwägung gezogen werden, um diese histopathologisch und mittels direkter Immunfluoreszenzmikroskopie zu untersuchen.

## FINANZIERUNG

Dieses Projekt wurde durch folgende Förderungen unterstützt: Rückkehrstipendium PR 1652/2‐1 (Deutsche Forschungsgemeinschaft [DFG]), Juniorförderung J03‐2020 (Universität zu Lübeck), Klinische Forschergruppe 303 (DFG), Sonderforschungsbereich 1526 (DFG) und Miniproposal 2021 (Exzellenzcluster 2167, DFG). J.P. erhielt zusätzlich Förderung durch das *Clinician Scientist Program* der Deutschen Stiftung Dermatologie e.V. in Zusammenarbeit mit der Deutschen Dermatologischen Gesellschaft e.V. (DDG; [https://derma.de/stipendien‐forschungspreise]) und der Arbeitsgemeinschaft Dermatologische Forschung e.V. (ADF; [https://www.adf‐online.de]). Das *Clinician Scientist Program* der DDG wurde freundlich unterstützt durch Abbvie Deutschland, Almirall, Janssen‐Cilag, LEO Pharma, Lilly, Sanofi und UCB Pharma.

## INTERESSENKONFLIKT

Die anderen Autoren haben keine Interessenkonflikte zu erklären.
